# Thin layer drying curves for shredded breadfruit (Artocarpus altilis)

**DOI:** 10.1111/jfpp.13146

**Published:** 2016-11-16

**Authors:** Camille George, Quinn Mogil, Michaela Andrews, George Ewing

**Affiliations:** ^1^ University of St. Thomas, 2115 Summit Ave St. Paul MN USA; ^2^ Compatible Technology International St. Paul MN USA

**Keywords:** breadfruit flour, fruits and vegetable drying, thin‐layer drying

## Abstract

A chart relating temperature and velocity to the drying time of single layered shredded breadfruit, Artocarpus altilis, is presented. Breadfruit, which can be dried and ground into gluten‐free flour, was placed on screens in a controlled drying chamber. A design of experiment based on the independent variables of temperature (21, 40, 55°C), absolute humidity (0.01, 0.01125, 0.0125 kg/kg dry air), and air velocity (2.5, 4, 6 m/s) was executed. The data suggested that the moisture ratio is exponentially related to the time spent drying, thus the Page model was used to analyze a response surface that identified the key factors that influence drying. An analysis of the response surface indicated that temperature, velocity, and initial moisture content are the critical drying factors. To enable drying predictions, two variable quadratic equations and a chart relating drying time to temperature and velocity, based on 10% moisture content as dry, are given.

**Practical applications:**

Breadfruit, Artocarpus altilis, is a fruit that grows in the tropical regions of the world. Drying breadfruit is a cost effective means of preserving this resource which can be ground into a gluten‐free flour. Processing breadfruit flour into nutritious products is an opportunity to overcome hunger, increase food security, and contribute to sustainable development on many island nations. Equations based on temperature and velocity and a chart relating these factors to an adequate drying time are presented. This original work provides new understandings of these variables and the chart relating drying time to air temperatures and velocities would be useful in post‐harvest economic trade‐off charts and system scaling.

**Nomenclature**
*MR*: moisture ratio
*k, n*: constants
*t*: time, min
*M*: moisture content
*m_w_*: mass of water, kg
*m_d_*: mass of dry matter, kg
*M_o_*: initial moisture content
*M_e_*: equilibrium moisture content
*T*: air temperature, °C
*v*: air velocity, m/s
*w*: absolute humidity, kg/kg dry air.

## 1 INTRODUCTION

Breadfruit, Artocarpus altilis, is a food that grows in the Caribbean and Pacific islands and can be consumed like a fruit or vegetable. Drying breadfruit significantly increases the shelf life of the produce and creates opportunities for byproducts such as gluten free breadfruit flour. Drying breadfruit is a cost effective means of preserving this underutilized resource. Understanding the factors that influence drying such as air temperature, air velocity, loading density, humidity, and produce shape and size can help determine more effective means of drying breadfruit.

Some authors claim that velocity has little effect on the drying rate of the produce while others incorporate velocity into their model as a significant factor (Akpinar & Yasar, 2005; Koyuncu, Tosun, & Ustun, 2003; Yaldýz & Ertekýn, 2001). This difference in effect is because air speed has a notable effect on drying if particular conditions are met. For a change in air speed to have a notable affect, the speed of the air needs to be below the minimum threshold for removing moisture, the product is spread as a deep bed instead of a thin layer, or if the product is spread on the trays in the direction of air flow (Driscoll, 2008).

The authors did not find any literature on the drying of breadfruit, but were able to find literature on the drying of other produce. Because the drying of most produce is in the falling rate period, nonlinear models are necessary to reflect the drying process (Akpinar & Yasar, 2005; Harrynanan & Sankat, 2005). There are different models which can be used to empirically model the drying of various kinds of produce including the Newton or Exponential, Page, Two‐Term, Logarithmic, Diffusion, and Modified Page models (Akin, Gurlek, & Ozbalta, 2014; Akpinar & Yasar, 2005; Basunia & Abe, 2001; Driscoll, 2008; Harrynanan & Sankat, 2005; Sacilik, 2007; Simal, Femenia, Garau, & Rosselló, 2005; Yaldýz & Ertekýn, 2001). The authors chose to focus on the Page model because it is a simple model and has successfully modeled the drying of produce such as rice and green beans (Afonso, Correa, & de Queiroz, 2001; Akpinar & Yasar, 2005; Basunia & Abe, 2001; Moreira, Chenlo, Torres, & Arufe, 2015) and various other kinds of produce (Akin et al., 2014; Akpinar & Yasar, 2005; Basunia & Abe, 2001; Sacilik, 2007; Simal et al., 2005; Yaldýz & Ertekýn, 2001). In the Page model the moisture ratio, or the percent moisture remaining relative to “dry” at particular conditions, is exponentially related to the time spent drying.

(1) $$MR\;=\;\text{exp}\left(-kt^{n}\right)$$

Although the Page model is an empirical model, the coefficients of the model have been compared to the drying factors (Afonso et al., 2001; Akpinar & Yasar, 2005; Basunia & Abe, 2001; Moreira et al., 2015; Simal et al., 2005). Some models relate the coefficients to factors such as temperature, humidity, and velocity through linear, quadratic, or exponential models. In some cases, these variables were not independent. For example, relative humidity is dependent on temperature and in a solar dryer, changing the velocity also changes the temperature.

This set of experiments will analyze the effect of temperature, absolute humidity, and air velocity as independent variables affecting drying time and the coefficients of the Page model. Initial moisture content was later added as a variable. The resultant equation can provide drying times for given conditions for system design and scale‐up.

## 2 MATERIAL AND METHODS

The three factors chosen for the design of experiment (DOE) are: temperature, absolute humidity, and air velocity. These factors had the largest impact on drying time in previous experiments. The DOE is a three‐factor, two‐level factorial, with a center point (italicized). Temperatures tested were 21, *40*, and 55°C, the absolute humidities tested were 0.01, *0.01125*, 0.0125 kg/kg dry air (9% to 75% relative humidity depending on temperature), and the air velocities tested were 2.5, *4*, and 6 m/s measured at the exit (0.020, 0.032, 0.049 m^3^/s). Absolute humidity is used as a variable instead of relative humidity to ensure that the experiment contained three independent variables.

Table 1 outlines the intended values of the factors for the experiment. The actual values during the experiment are listed in Table 2. Initial moisture content was added as a variable and back calculated after the experiments to determine if this had an effect on the drying curves in addition to the intended variables. The initial moisture content can vary with the variety and ripeness of the breadfruit. Different varieties of breadfruit were used throughout the experiment. The actual values for the variables in addition to the initial moisture content were used in the response surface analysis.

**Table 1:** Intended experimental conditions

Standard order	Run order	Temperature (°C)	Absolute humidity (kg/kg)	Relative humidity (%)	Air velocity (m/s)
**1**	12	21	0.01	64	6
**2**	2	55	0.01	10	2.5
**3**	7	21	0.0125	80	2.5
**4**	9	55	0.01	10	6
**5**	4	21	0.01	64	2.5
**6**	6	55	0.0125	12.7	6
**7**	3	21	0.0125	80	6
**8**	5	55	0.0125	12.7	2.5
**9**	8	40	0.01125	35.5	4
**10**	11	40	0.01125	35.5	4
**11**	1	40	0.01125	35.5	4
**12**	10	40	0.01125	35.5	4

**Table 2:** Actual experimental conditions from run data

Standard order	Tc avg	*w* avg	*v* avg (m/s)	MC o
1	22.2	0.0102	6.03	76.20%
2	49.5	0.0081	2.66	85.10%
3	21.2	0.013	2.6	71.40%
4	48.4	0.0093	5.99	72.50%
5	21.8	0.0098	2.77	75.60%
6	45.6	0.0106	6.11	80.20%
7	20.1	0.0109	5.96	74.30%
8	49.8	0.0111	2.51	83.80%
9	41.3	0.0097	4.44	78.10%
10	42.8	0.0095	4.17	73.40%
11	41.4	0.0098	4.51	85.40%
12	42.9	0.0094	4.23	72.50%

### 2.1 Equipment

The equipment used in the experiments are listed in Table 3.

**Table 3:** Equipment used for this experiment

Instrument	Manufacturer and model	Resolution	Accuracy	Purpose in experiment
**Hygrometer**	Omega: Omegette HH311	0.1%RH	±2.5%RH at 25°C ±0.7°C	Humidity and temperature measurements
**Hand‐held vane anemometer**	Alnor: RVA801	0.01 m/s	±1% of reading 0.02 m/s	Velocity measurements
**Digital psychrometer**	General Tools: EP8706	T(°F): 0.1°F %RH: 0.1%RH	±1°F ±3%RH at 25°C	Ambient condition measurement
**Digital scale**	Tree: HRB‐3002	0.01 g	±0.02 g repeatability	Mass measurements

### 2.2 Experimental Setup

**Figure 1:**
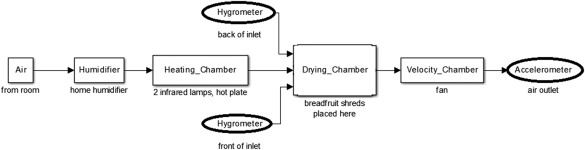
Flow chart of drying process

**Figure 2:**
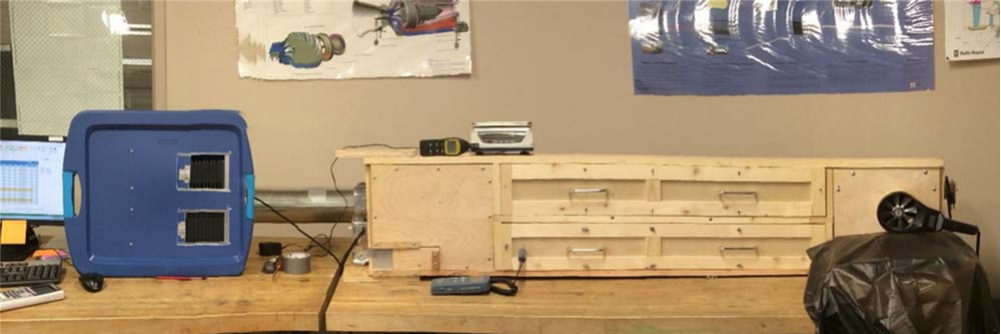
Image of experimental setup

Ambient air was pulled through conditioning chambers prior to the single‐pass tray dryer to achieve the run conditions. Figure 1 shows the order of these conditioning chambers and Figure 2 is an image of the experimental setup. The first conditioning chamber consisted of a housing containing a humidifier. This connected to a heating chamber with two infrared lamps and an adjustable hot plate. The air was pulled across the drying trays by a fan located in the velocity chamber. Three drying trays were used, each with 0.07 m^2^ drying surface area. The trays had aluminum bar stock frames with landscape fabric sewn around the frames to hold the breadfruit shreds. The ambient air conditions in the room were read with a digital psychrometer. The dry bulb temperature and relative humidity of the air were read at the front and back of the inlet of the tray chamber using hygrometers. The air velocity was read using a hand‐held vane anemometer at the outlet of the velocity chamber.

### 2.3 Experimental procedure

The breadfruit selected for the experiment was firm and did not yield when pressed by hand. The breadfruit was peeled, cored, and shredded into 6 mm shreds using a Cuisinart food processor. The breadfruit shreds were then spread evenly on each of the three trays with a loading density of 1.6 kg/m^2^. The heating, humidifying, and velocity conditions were setup and the chamber given time to reach system steady state.

The trays with the shreds were placed in the chamber and the mass of the breadfruit shreds and chamber conditions were recorded initially at 5‐min intervals, then 10‐min intervals for 2 hr, then 20‐min intervals for 2 hr, and then 30 min intervals until the end of the experiment. The conditions recorded include the humidity, the outlet temperature, and the velocity. Each run concluded when the change in mass of the breadfruit was a fraction of a gram for three readings. A sample of the dried shreds was sealed in a plastic bag to be sent to a commercial laboratory for moisture content analysis by oven and vacuum.

## 3 THEORY/CALCULATION

The moisture content was determined using the wet basis calculation

(2) $$M=m_{w}/\left(m_{w}+m_{d}\right)$$

where *M* is the moisture content at a given time, *m_w_* is the mass of the moisture, and *m_d_* is the mass of the dry matter (Driscoll, 2008). The equilibrium moisture content was determined by oven and vacuum at a commercial laboratory. According to Driscoll, Fick's diffusion equation to moisture ratio can be related to exponential decay

(3) $$MR=(M-M_{e})/(M_{o}-M_{e})=exp(-kt^{n})$$

where *MR* is the moisture ratio, *M* is the moisture content at time *t*, *M_e_* is the equilibrium moisture content, and *M_o_* is the initial moisture content (Driscoll, 2008). Some authors simplify this to *M*/*M*_o_ because of varying humidity, but because humidity is constant for each test in this experiment the full moisture ratio equation can be used (Akin et al., 2014; Sacilik, 2007).

**Figure 3:**
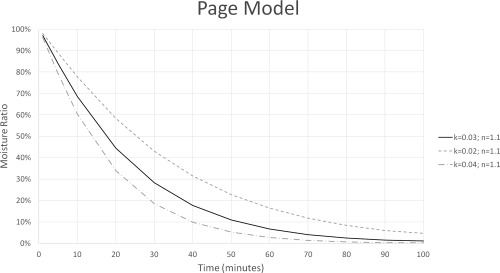
This displays how changing *k* affects the model of the drying curve

**Figure 4:**
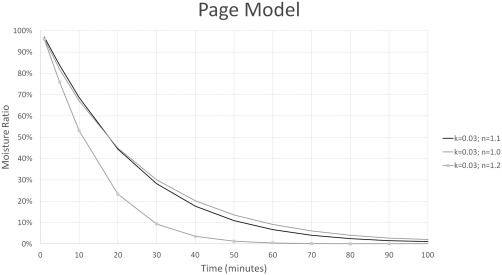
This displays how changing *n* affects the model of the drying curve

**Figure 5:**
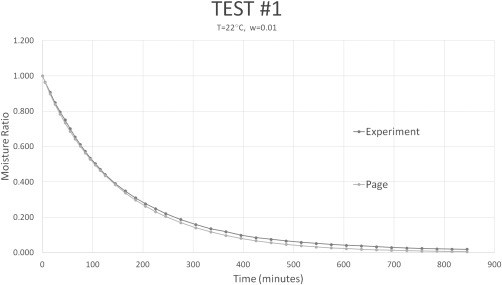
The Page model fit to the moisture ratio data from test #1. The Pearson number for this fit was 0.9999

The Page model was fit to each of the tests using Microsoft Excel Solve to determine the constants *k* and *n* for each experiment. Both *k* and *n* affect the shape of the drying curve and therefore affect the predicted drying time. Even a small change in one of these constants can greatly impact drying time. For example, changing *n* from 1.1 to 1.2 increases the time it takes for the moisture ratio to be below 1% by 1 hr. Figures 3 and 4 illustrate how changing the value of *k* or *n* by 0.01 or 0.1, respectively can significantly impact the drying curve. This contradicts claims that *n* can be assumed constant because it changed little with loading density or temperature (Moreira et al., 2015; Simal et al., 2005). Figure 4 shows that even a small change in the value of *n* can greatly impact the drying curve. Table 4 lists the fitted Page model constants and statistics confirming that the Page models fit the experimental data well. Figure 5 presents a typical drying curve from experimental data and a fitted drying curve based on the Page model.

**Table 4:** List of tests with corresponding Page model constants, *k* and *n*, determined through fitting the curve to the experimental moisture ratio data

Test	k Experiment model	n Experiment model	Page Pearson number	Page RMSE (kg/kg)	Page Chi‐Sq
1	0.0079	0.9628	0.9999	0.015	0.283
2	0.0087	1.2806	0.9998	0.008	0.233
3	0.0016	1.1033	0.9996	0.028	0.171
4	0.0335	1.008	0.9998	0.006	0.016
5	0.0038	1.13	0.9999	0.010	0.026
6	0.0421	1.038	0.9996	0.009	0.010
7	0.0048	1.0592	1	0.006	0.018
8	0.0105	1.2528	0.9994	0.014	0.013
9	0.0096	1.2675	0.9997	0.010	0.009
10	0.0127	1.1192	1	0.003	0.004
11	0.0107	1.2444	0.9999	0.004	0.037
12	0.0153	1.0774	0.9996	0.012	0.251

The Pearson number gives an indication of how good the fit is; the closer to 1.0 the better (Lane, n.d.). The RMSE value indicates that the moisture ratio had a maximum error of 0.028, which is 5.5% of the average moisture ratio value for that experiment. Lastly, the Chi‐Squared values are all well below the critical values corresponding to the degrees of freedom in each test (Kernler, n.d.).

The constants *k* and *n* were then put into the Minitab DOE as the results and the DOE was analyzed to determine which factors most influenced the constants of the Page model (Minitab, Inc., 2014). In this experiment, temperature and velocity most affected *k*, and velocity and initial moisture content most affected *n*.

## 4 RESULTS AND DISCUSSION

As indicated by other authors, the Page model works well as an empirical model for drying. The Pearson number gives an indication of how good the fit is; the closer to ±1.0 the better. A Pearson value of 0 would indicate no correlation. The Pearson correlation value was above 0.999 for all twelve experiments. This indicates an excellent fit between the Page model and the experimental values. Other statistics such as the RMSE and Chi‐Squared also indicated an excellent fit between the experimental drying curve and the modeled drying curve.

The values for *k* and *n* were then put as the results for the Minitab Response Surface Analysis. The analysis indicated that temperature and velocity are the factors that most influence *k*, and velocity and initial moisture content are the factors which most influence *n*. Tables 5 and 6 give the results of the Minitab Response Surface analysis which was indicated by low *p* values for these factors. A low *p* value indicates that the factor is significant. A high F value also indicates that a factor is significant (Anon, 2015).

**Table 5:** Minitab response surface analysis of what factors most influence the constant *k*

Source	F value	*p* value
***T* avg (C)**	66.93	0
***v* avg (m/s)**	48.35	0
Square: v*v	14.14	.007
2‐way interaction: T*v	27.94	.001

**Table 6:** Minitab response surface analysis of what factors most influence the constant *n*

Source	F value	*p* value
***v* avg (m/s)**	14.41	.005
**MC_0_**	17.91	.003
Square: v*v	6.6	.033

**Figure 6:**
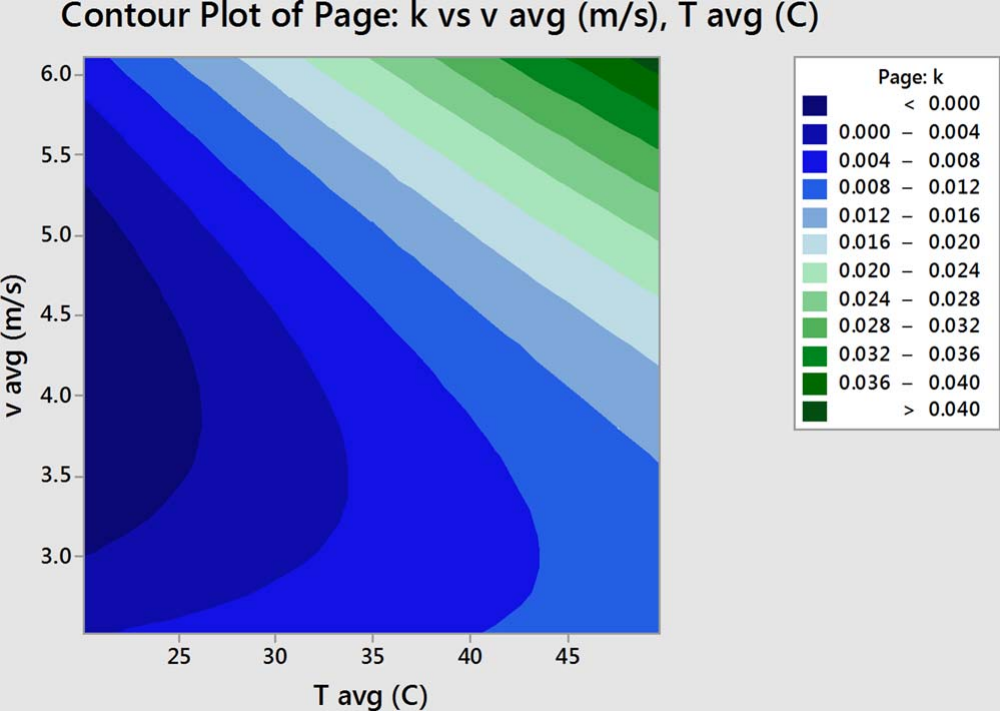
Contour plot of the effect of air temperature and velocity on *k*

**Figure 7:**
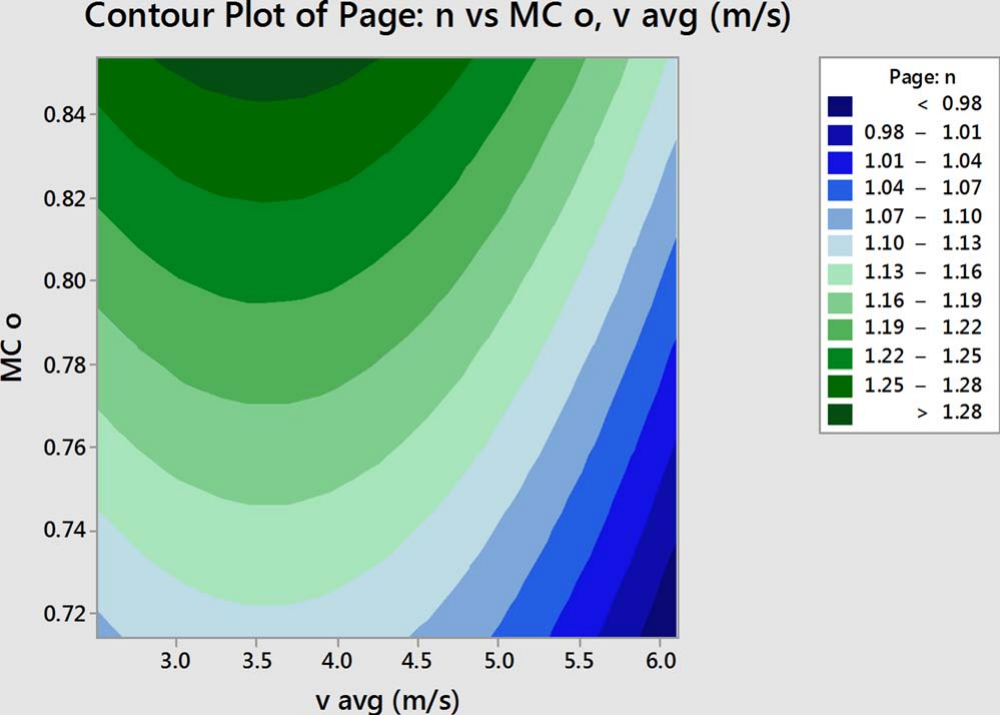
Contour plot of the effect of initial moisture content and velocity on *n*

Using the response surface analysis, coefficients could be determined relating the main factors, temperature, velocity, and initial moisture content, to the Page model constants *k* and *n*. Contour plots present a graphical representation of these two variable equations. Figure 6 presents the contour plot for *k*, and Figure 7 presents the contour plot for *n*.

(4) $$k=0.0535-0.000481*T-0.02836*v+0.00274*v^{2}+0.000274*T*v$$

(5) $$n=-0.093+1.235*MC_{0}+0.1864*v-0.262*v^{2}$$

Lastly, the factors of temperature and velocity can be directly related to drying time through assuming an initial moisture content of 75% and considering a moisture ratio of 10% to be dry. This leaves time related to only temperature and velocity. With these assumptions, the Page model equation can be solved for time, as shown in *Equation* (6),

(6) t=−1k*ln⁡MR1n

where *k* remains a function of temperature and velocity as shown in *Equation* (4). Given MC_0_ = 75%, the *n* is only a function of velocity and *Equation* (5) can be simplified to *Equation* (7).

(7) $$n=0.833+0.1864*v-0.0262*v^{2}$$

**Figure 8:**
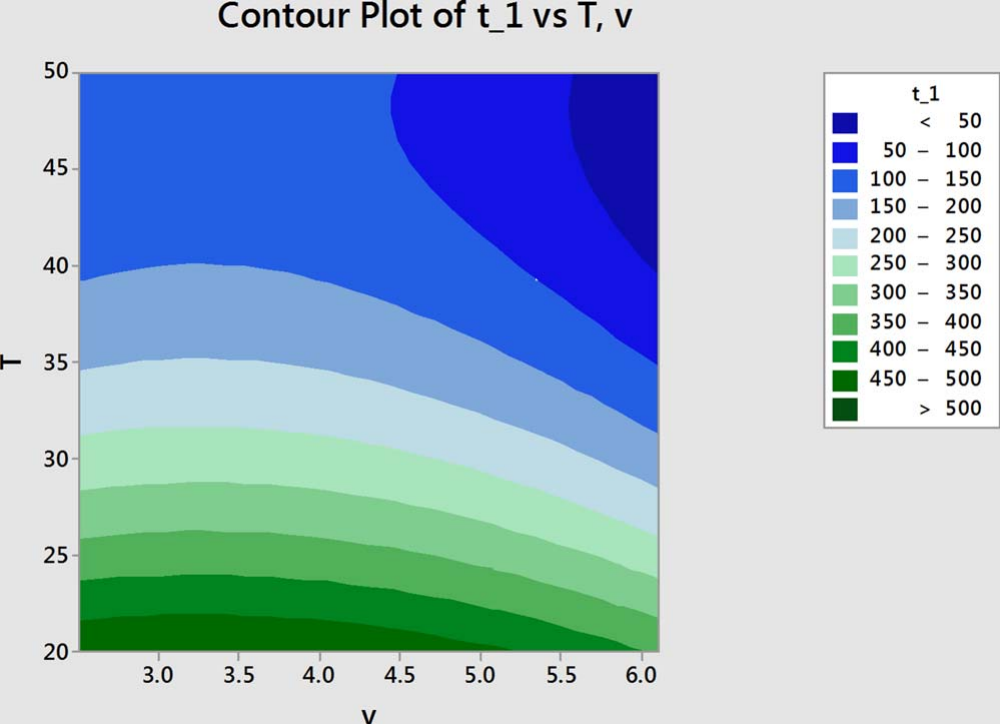
Response surface depicting the effect of temperature and air velocity on drying time. Drying time for a thin layer of breadfruit can be estimated if the temperature and air velocity are known

*Equations* (4) and (7) for *k* and *n* can be inserted into their respective places in *Equation* (6), therefore making drying time a function of temperature and velocity. Figure 8 is an empirical representation of that model based on the data collected. The R‐squared value, which ranges from 0 to 100% with 100% being a perfect fit, was 84% for this model. This indicates that the model adequately fits the data. The R‐squared predicted value, which indicates how well the model can predict values outside of the data set, is 77% for this model. This also indicates that this model adequately depicts how air temperature and velocity affect drying time. Given air temperature and velocity, thin layer breadfruit drying time can be approximated through the response surface.

## 5 CONCLUSIONS

In this experiment the Page model was used to empirically model the drying of shredded breadfruit, Artocarpus altilis, under varying temperature, air velocity, humidity, and initial moisture content. In this experiment, the most significant factors were temperature, air velocity, and initial moisture content.

This experiment confirms that *n* varies little with temperature and that it is affected by air velocity and initial moisture content (Moreira et al., 2015; Simal et al., 2005). It also confirms that *k* increases with increasing temperature, but does not confirm that it is a function of initial moisture content (Moreira et al., 2015; Simal et al., 2005). This study confirms that velocity is a significant factor if the product is on trays in the direction of airflow (Driscoll, 2008). Although some authors claim that *k* and *n* are both functions of temperature and relative humidity or temperature and velocity, this experiment found that velocity is a factor for both but temperature is only a factor for *k* and relative humidity is not a factor for either (Afonso et al., 2001; Akpinar & Yasar, 2005; Basunia & Abe, 2001). By relating temperature and velocity to drying time, a contour plot indicated that temperature is the primary factor for drying time. Future studies could explore other factors and techniques such as loading density or pretreatments.

## ORCID

*Camille George* http://orcid.org/0000-0001-8503-2420
